# Severe Oral Mucositis After Intensity-Modulated Radiation Therapy for Head and Neck Cancer

**DOI:** 10.1001/jamanetworkopen.2023.37265

**Published:** 2023-10-11

**Authors:** Austin J. Iovoli, Lauren Turecki, Michelle L. Qiu, Michael Khan, Kelsey Smith, Han Yu, Sung Jun Ma, Mark K. Farrugia, Anurag K. Singh

**Affiliations:** 1 Department of Radiation Medicine, Roswell Park Comprehensive Cancer Center, Buffalo, New York; 2 currently a medical student at Jacobs School of Medicine and Biomedical Sciences, University at Buffalo, Buffalo, New York; 3 Department of Biostatistics & Bioinformatics, Roswell Park Comprehensive Cancer Center, Buffalo, New York; 4 currently affiliated with Department of Radiation Oncology, The Arthur G. James Cancer Hospital and Richard J. Solove Research Institute, The Ohio State University Comprehensive Cancer Center, Columbus, Ohio

## Abstract

**Importance:**

Oral mucositis (OM) is a common and debilitating adverse effect observed in patients with head and neck cancer (HNC) receiving radiation therapy (RT). Previous studies examining associations between OM and clinical outcomes were performed in the era of 3-dimensional conformal RT planning with low rates of concurrent chemotherapy, and thus may not reflect current practice.

**Objective:**

To prospectively assess patient-reported OM and identify its associations with clinical outcomes and quality of life.

**Design, Setting, and Participants:**

This cohort study performed at a single institution included 702 consecutive patients who underwent definitive or adjuvant intensity-modulated RT (IMRT) for primary HNC from February 9, 2015, to May 27, 2022. Data were analyzed from November 28, 2022, to August 18, 2023.

**Main Outcomes and Measures:**

Severity of OM was assessed based on highest reported mouth and throat soreness (MTS) score during radiotherapy according to the Oral Mucositis Weekly Questionnaire–Head and Neck Cancer survey, which was administered weekly during IMRT. Linear mixed models were used to compare mean MTS scores grouped by disease site and chemotherapy regimen. Fisher exact tests and 1-way analysis of variance tests were performed to identify associations between severity of OM and clinical outcomes.

**Results:**

Among 576 eligible patients, the median age was 62.5 (IQR, 56.3-69.1) years, and 451 patients (78.3%) were men. In terms of race and ethnicity, 6 patients (1.0%) were American Indian or Alaska Native; 2 (0.3%), Asian; 31 (5.4%), Black; 8 (1.4%), Hispanic or Latino; 509 (88.4%), White; and 28 (4.9%), unknown. The most common treatment site was oropharynx (268 [46.5%]), and most patients received concurrent chemotherapy (464 [80.6%]). By the end of treatment, 360 patients (62.5%) developed severe OM and 568 (98.6%) developed some degree of OM. Linear mixed models found no significant differences in OM between HNC disease sites. Groups with greater highest severity of OM reported had higher rates of measured outcomes (listed respectively by MTS score 0, 1, 2, 3, and 4): feeding tube placement (0%, 3.6% [2 of 56], 6.6% [10 of 152], 14.7% [40 of 272], and 21.6% [19 of 88]; *P* = .001), hospitalization (12.5% [1 of 8], 10.7% [6 of 56], 15.1% [23 of 152], 23.9% [65 of 272], and 28.4% [25 of 88]; *P* = .02), opiate use (0%, 19.6% [11 of 56], 42.8%[65 of 152], 61.4% [167 of 272], and 64.8% [57 of 88]; *P* < .001) and experienced greater weight loss (median, −0.7 [IQR, −1.7 to −0.4] kg; median, 3.9 [IQR, 1.1 to 6.1] kg; median, 5.0 [IQR, 2.2 to 7.7] kg; median, 4.7 [IQR, 2.1 to 7.7] kg; and median, 7.7 [IQR, 2.8 to 10.6] kg; *P* < .001).

**Conclusions and Relevance:**

In this cohort study of patients with HNC, 62.5% developed severe OM. Higher severity of OM was associated with feeding tube placement, hospitalization, opiate use, and weight loss. Improvements in OM prevention and management are needed.

## Key Points

**Question:**

In patients undergoing radiation therapy for head and neck cancer, what factors are associated with higher-severity oral mucositis, and what clinical outcomes follow?

**Findings:**

In this cohort study of 576 patients, 360 (62%) developed severe oral mucositis. This was associated with feeding tube placement, hospitalization, weight loss, and opiate use.

**Meaning:**

These findings suggest that oral mucositis continues to cause morbidity in patients with head and neck cancer, contributing to worse quality of life and financial effects.

## Teaser

This cohort study describes patient-reported oral mucositis and identifies associations with clinical outcomes among patients with head and neck cancer.

## Introduction

Oral mucositis (OM) is a common and debilitating adverse effect observed in patients with head and neck cancer (HNC) receiving radiation therapy (RT).^1^ It is characterized by painful inflammation and ulceration of the mucosal membranes in the mouth and throat. This negatively affects patients by causing pain and discomfort while also diminishing the ability to speak, swallow, and eat.^1,2^ The sequelae of OM have been shown to increase opioid use, weight loss, feeding tube placement, and hospitalization among patients with HNC.^3,4,5,6^ These symptoms put patients at risk for treatment interruptions that can compromise clinical outcomes.^7^

Despite the significant clinical effect OM has on patient care, there is no single agreed-upon method for measuring its severity, and multiple tools have been reported.^8^ Previous studies^3,4,5,6^ have used these tools to examine the link between OM severity and patient-reported quality of life (QOL); however, these studies may not reflect current practice, as they were performed predominantly in the era of less precise 3-dimensional conformal RT planning and less frequent use of concurrent chemotherapy. Modern RT planning for HNC is routinely performed with intensity-modulated RT (IMRT), which allows for better sparing of normal structures than previous techniques. The use of IMRT in HNC is associated with reductions in xerostomia, dysphagia, and effects in multiple QOL domains.^9,10,11,12^

The effect that modern treatment planning with IMRT has on acute OM symptoms and QOL during RT for HNC is not well characterized. We performed a contemporary update using validated questionnaires in a large cohort of patients with HNC to provide a detailed description of patient-reported OM and identify associations with clinical outcomes.

## Methods

### Study Population

The patient population of this cohort study was derived from a collection of 702 consecutive patients who underwent definitive or adjuvant RT for primary HNC at a single center from February 9, 2015, to May 27, 2022. To be included in this study, patients had to meet the following criteria: (1) pathologically confirmed primary HNC, (2) received treatment with definitive or adjuvant intent RT, (3) completed RT, and (4) at least 6 weeks of OM data available. A total of 576 patients met inclusion criteria (eFigure in Supplement 1). Data were collected under a protocol approved by the Institutional Review Board at Roswell Park Comprehensive Cancer Center. Informed consent was waived since our study met the criteria for minimal risk to the study participants. The Strengthening the Reporting of Observational Studies in Epidemiology (STROBE) reporting guideline was followed.

### Patient Treatment and Medical Record Abstraction

Staging workup was completed in all patients with computed tomography of the head and neck with contrast and/or positron emission tomography–computed tomography. All patients underwent IMRT (60-70 Gy for 30-35 fractions to the primary tumor, 54-56 Gy for 30-35 fractions to elective lymph nodes), with or without concurrent chemotherapy, as previously described.^13^ For patients treated with concurrent chemotherapy, cisplatin, 100 mg/m^2^, was intravenously infused once every 3 weeks or cisplatin, 40 mg/m^2^, was administered intravenously once weekly. The first chemotherapy dose began with day 1 of RT. Pertinent clinicopathologic data were abstracted from the medical record and stored in a secure Research Electronic Data Capture (REDCap) database.^14,15^ Race and ethnicity data were obtained from the medical record, which was entered at the time of initial registration based on patient self-identification. For assessment of clinical outcomes, patient hospitalization and nonprophylactic feeding tube placement were defined as events occurring within 100 days of starting RT.

Prior to and during treatment, all patients received educational materials and encouragement about oral hygiene, hydration, and nutrition. Patients were encouraged to gargle with a saline and baking soda mouthwash rinse as many times as possible per day (eg, 20 times) and use a compounded elixir of diphenhydramine, xylocaine, and antacid in a 1:1:1 ratio 4 times per day for pain. Further details of our institutional management of OM have been previously reported.^16^

As a part of routine institutional practice, patients were evaluated weekly while undergoing RT by the radiation clinical team through physical examination and patient-reported responses to a modified Oral Mucositis Weekly Questionnaire–Head and Neck Cancer (OMWQ-HN) survey (eAppendix in Supplement 1).^17^ The OMWQ-HN is a valid and reliable survey assessing patient well-being and function. Physical examination included assessment of OM, weight changes, and feeding tube use. Development of severe OM was defined as “quite a lot” or “extreme” reported for the OMWQ-HN mouth and throat soreness (MTS) item. Patients were grouped as developing severe OM category based on their highest reported MTS score (range, 0 [no OM] to 4 [most severe OM]). The decision for feeding tube placement was performed with multidisciplinary evaluation of factors, including patients’ nutritional and functional status, speech and swallow evaluation, and shared discussions among patients, family members, caregivers, and physicians. Patient-reported QOL was assessed on the first and last day of RT with the European Organisation for Research and Treatment of Cancer Core Quality of Life Questionnaire.^18,19^ Figures based on the OMWQ-HN and clinical data were generated using Excel for Microsoft 365 MSO, version 2205 Build 16.0.15225.20362 (Microsoft Corporation).

### Statistical Analysis

Patient baseline demographic and clinical information were summarized using frequency (percentages) and medians (IQRs). Associations between clinical characteristics and highest patient-reported severity of OM during RT were assessed using Fisher exact tests and 1-way analysis of variance, as appropriate. For longitudinal assessment of OM development, linear mixed models were used to compare mean MTS scores grouped by disease site and chemotherapy regimen. Model fit was by the restricted maximum likelihood method. A model with time × group interaction effect was fit first, and if the interaction effect was not statistically significant, the model with main effects was used. Clinical outcomes such as feeding tube placement, hospitalization, opiate use, and weight loss were reported by week of RT and associated with highest patient-reported severity of OM using Fisher exact tests and analysis of variance, as appropriate.

Associations between development of severe OM and baseline QOL were assessed using the Mann-Whitney test. This method was also used to assess associations between development of severe OM and change in QOL score from the start to end of RT. For all analyses, 2-sided *P* < .05 was considered statistically significant. Analyses were performed using R, version 4.2.0 (R Project for Statistical Computing). Data were analyzed from November 28, 2022, to August 18, 2023.

## Results

Among 576 eligible patients, the median age was 62.5 (IQR, 56.3-69.1) years; 451 patients (78.3%) were men and 125 (21.7%) were women. In terms of race and ethnicity, 6 patients (1.0%) were American Indian or Alaska Native; 2 (0.3%), Asian; 31 (5.4%), Black; 8 (1.4%), Hispanic or Latino; 509 (88.4%), White; and 28 (4.9%), unknown. The most common site treated was the oropharynx (268 [46.5%]), and most patients received concurrent chemotherapy (464 [80.6%]). Data from the OMWQ-HN were available for 576 patients for weeks 1 to 6 of RT and for 466 patients for week 7. Complete patient demographic and treatment information is described in Table 1.

**Table 1.** Patient Characteristics of the Total Cohort That Received RT for Head and Neck Cancer^a^

Characteristic	Patient data (n = 576)
Age, median (IQR), y	62.5 (56.3-69.1)
Sex	
Men	451 (78.3)
Women	125 (21.7)
Race	
American Indian or Alaska Native	6 (1.0)
Asian	2 (0.3)
Black	31 (5.4)
White	510 (88.5)
Unknown	27 (4.7)
Ethnicity	
Hispanic or Latino	8 (1.4)
Non-Hispanic or Latino	549 (95.3)
Unknown	19 (3.3)
ECOG performance status^b^	
0	368 (63.9)
1	136 (23.6)
2	66 (11.5)
3	6 (1.0)
BMI, median (IQR)	27.8 (24.2-31.9)
Primary site	
Oropharynx	268 (46.5)
Nasopharynx	21 (3.6)
Hypopharynx	27 (4.7)
Larynx	118 (20.5)
Oral cavity	78 (13.5)
Parotid gland	17 (3.0)
Unknown	47 (8.2)
Stage	
I	82 (14.2)
II	67 (11.6)
III	130 (22.6)
IV	297 (51.6)
HPV status	
Positive	265 (46.0)
Negative	106 (18.4)
Unknown	205 (35.6)
Treatment type	
RT alone	48 (8.3)
ICT plus CCRT	38 (6.6)
CCRT	344 (59.7)
Surgery plus RT	62 (10.8)
Surgery plus CCRT	84 (14.6)
Chemotherapy regimen	
Cisplatin every 21 d	328 (56.9)
Cisplatin weekly	54 (9.4)
Noncisplatin regimen	82 (14.2)
No chemotherapy	112 (19.4)
Neck RT	
Unilateral	157 (27.3)
Bilateral	398 (69.1)
NA	21 (3.6)
Smoking status	
Never	160 (27.8)
Former or current	416 (72.2)

Abbreviations: BMI, body mass index (calculated as weight in kilograms divided by height in meters squared); CCRT, concurrent chemoradiation; ECOG, Eastern Cooperative Oncology Group; HPV, human papillomavirus; ICT, induction chemotherapy; NA, not available; RT, radiation therapy.

^a^ Unless otherwise indicated, data are expressed as No. (%) of patients. Percentages have been rounded and may not total 100.

^b^ Scores range from 0 (no restrictions) to 5 (death).

### OMWQ-HN Outcomes

**Figure 1.**
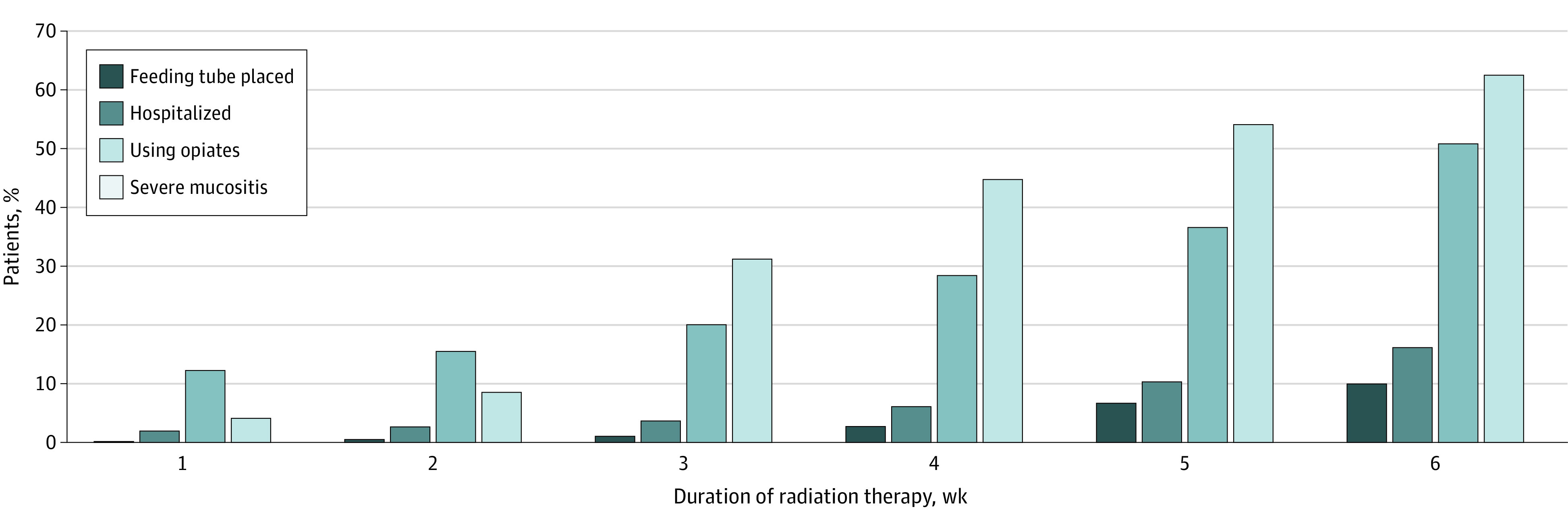
Cumulative Incidence of Feeding Tube Placement, Hospitalization, Opiate Use, and Severe Oral Mucositis for 576 Patients With Head and Neck Cancer Undergoing Radiation Therapy

During the course of RT, 360 patients (62.5%) developed severe OM (Figure 1). The largest increase in patients developing severe OM was from 49 (8.5%) to 180 (31.3%) between weeks 2 and 3 of treatment. Most patients (568 [98.6%]) developed some degree of MTS by week 7 of treatment. Increases in MTS during RT contributed to patient-reported functional declines in swallowing, drinking, eating, and talking. By the final week of RT, at least 50% of patients reported moderate or greater limitations in swallowing (327 [56.8%]), eating (400 [69.4%]), and drinking (280 [48.6%]). Three hundred fifty-eight of 466 patients (76.8%) were limited to a pureed diet and/or feeding tube for nutrition at the end of RT. Functional limitations contributed to a mean (SD) patient-reported overall health rating decline of 1.8 (1.7) points to a score of 5.9 (2.2) of 10 at the end of RT. Complete OMWQ-HN results by week of RT are summarized in eTable 1 in Supplement 1.

### OM Risk

**Figure 2.**
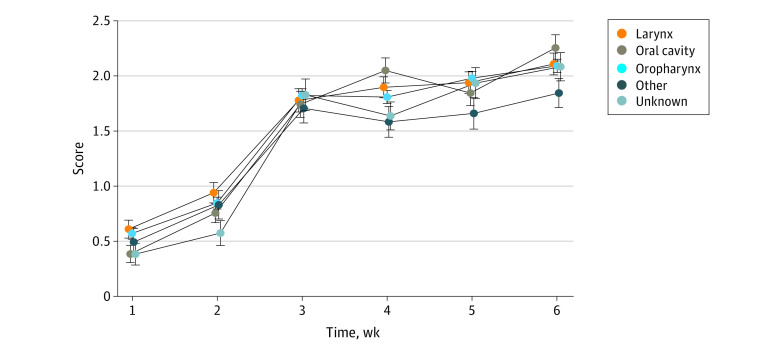
Linear Mixed Model of Mean Mouth and Throat Soreness Scores by Primary Head and Neck Cancer Disease Site Scores range from 0 to 4, with higher scores indicating greater severity of oral mucositis.

The distribution of patient clinical characteristics grouped by highest severity of patient-reported OM is summarized in Table 2. Factors associated with severity of OM included primary disease site, treatment type, bilateral neck coverage, and former or current smoking status. Longitudinal assessment of the data with linear mixed models of mean MTS scores by disease site found no significant differences between sites (Figure 2). There was no association between different chemotherapy regimens and OM severity. Furthermore, linear mixed models of mean MTS scores found no significant difference between patients receiving no chemotherapy and those receiving weekly cisplatin (β = −1.10 ± .11; *P* = .35) or cisplatin every 21 days (β = 0.13 ± .07; *P* = .07).

**Table 2.** Patient Characteristics of the Total Cohort Grouped by Highest Severity of Patient-Reported MTS During RT for Head and Neck Cancer^a^

Characteristic	MTS score^b^					*P* value
	0 (n = 8)	1 (n = 56)	2 (n = 152)	3 (n = 272)	4 (n = 88)	
Age, median (IQR), y	74.9 (62.7-81.6)	63.2 (56.8-68.4)	62.8 (56.3-69.9)	61.9 (56.1-68.4)	62 (56.2-70.0)	.17
Sex						
Men	7 (1.6)	44 (9.8)	126 (27.9)	213 (47.2)	61 (13.5)	.18
Women	1 (0.8)	12 (9.6)	26 (20.8)	59 (47.2)	27 (21.6)	
Race						
American Indian or Alaska Native	1 (16.7)	0	2 (33.3)	2 (33.3)	1 (16.7)	.33
Asian	0	0	1 (50.0)	1 (50.0)	0	
Black	0	3 (9.7)	11 (35.5)	11 (35.5)	6 (19.4)	
White	7 (1.4)	48 (9.4)	134 (26.3)	242 (47.5)	79 (15.5)	
Unknown	0	5 (18.5)	4 (14.8)	16 (59.3)	2 (7.4)	
Ethnicity						
Hispanic or Latino	0	2 (25.0)	1 (12.5)	4 (50.0)	1 (12.5)	.50
Non-Hispanic or Latino	8 (1.5)	50 (9.1)	148 (27.0)	259 (47.2)	84 (15.3)	
Unknown	0	4 (21.1)	3 (15.8)	9 (47.4)	3 (15.8)	
ECOG performance status^b^						
0	4 (1.1)	41 (11.1)	101 (27.4)	167 (45.4)	55 (14.9)	.55
1	1 (0.7)	10 (7.4)	33 (24.3)	70 (51.5)	22 (16.2)	
2	3 (4.5)	4 (6.1)	17 (25.8)	31 (47.0)	11 (16.7)	
3	0	1 (16.7)	1 (16.7)	4 (66.7)	0	
BMI, median (IQR)	25.3 (23.3-29.6)	27.6 (24.2-32.3)	27.3 (24.1-31.8)	27.9 (24.2-32.0)	28.7 (24.4-32.6)	.38
Primary site						
Oropharynx	0	30 (11.2)	78 (29.1)	117 (43.7)	43 (16.0)	.01
Nasopharynx	0	2 (9.5)	3 (14.3)	14 (66.7)	2 (9.5)	
Hypopharynx	1 (3.7)	1 (3.7)	7 (25.9)	13 (48.1)	5 (18.5)	
Larynx	2 (1.7)	11 (9.3)	25 (21.2)	58 (49.2)	22 (18.6)	
Oral cavity	1 (1.3)	7 (9.0)	16 (20.5)	43 (55.1)	11 (14.1)	
Parotid gland	4 (23.5)	1 (5.9)	4 (23.5)	7 (41.2)	1 (5.9)	
Unknown	0	4 (8.5)	19 (40.4)	20 (42.6)	4 (8.5)	
Stage						
I	0	8 (9.8)	29 (35.4)	34 (41.5)	11 (13.4)	.48
II	0	7 (10.4)	15 (22.4)	30 (44.8)	15 (22.4)	
III	2 (1.5)	9 (6.9)	29 (22.3)	68 (52.3)	22 (16.9)	
IV	6 (2.0)	32 (10.8)	79 (26.6)	140 (47.5)	40 (13.6)	
HPV status						
Positive	0	31 (11.7)	73 (27.5)	124 (46.8)	37 (14.0)	.12
Negative	2 (1.9)	7 (6.6)	31 (29.2)	50 (47.2)	16 (15.1)	
Unknown	6 (2.9)	18 (8.8)	48 (23.4)	98 (47.8)	35 (17.1)	
Treatment type						
RT alone	0	2 (4.2)	14 (29.2)	25 (52.1)	7 (14.6)	.006
ICT plus CCRT	0	1 (2.6)	6 (15.8)	20 (52.6)	11 (28.9)	
CCRT	1 (0.3)	35 (10.2)	92 (26.7)	158 (45.9)	58 (16.9)	
Surgery plus RT	5 (8.1)	7 (11.3)	15 (24.2)	29 (46.8)	6 (9.7)	
Surgery plus CCRT	2 (2.4)	11 (13.1)	25 (29.8)	40 (47.6)	6 (7.1)	
Chemotherapy regimen						
Cisplatin every 21 d	1 (0.3)	32 (9.8)	84 (25.6)	162 (49.4)	49 (14.9)	.08
Cisplatin weekly	1 (1.9)	7 (13.0)	21 (38.9)	19 (35.2)	6 (11.1)	
Noncisplatin regimen	1 (1.2)	7 (8.5)	18 (22.0)	37 (45.1)	19 (23.2)	
Neck RT						
Unilateral	5 (3.2)	23 (14.6)	42 (26.8)	71 (45.2)	16 (10.2)	.002
Bilateral	2 (0.5)	1 (0.3)	105 (28.6)	189 (51.5)	70 (19.1)	
Smoking status						
Never	4 (2.5)	23 (14.4)	45 (28.1)	63 (39.4)	25 (15.6)	.04
Former or current	4 (1.0)	33 (7.9)	107 (25.7)	209 (50.2)	63 (15.1)	

Abbreviations: BMI, body mass index; CCRT, concurrent chemoradiation; ECOG, Eastern Cooperative Oncology Group; HPV, human papilloma virus; ICT, induction chemotherapy; MTS, mouth and throat soreness; RT, radiation therapy.

^a^ Unless otherwise indicated, data are expressed as No. (%) of patients in each row. Percentages have been rounded and may not total 100.

^b^ Higher scores indicate greater severity.

### Clinical Outcomes

The frequency of selected clinical outcomes grouped by highest severity of patient-reported OM is summarized in Table 3 and cumulative incidence of clinical outcomes by week of treatment is shown in Figure 1. A feeding tube was placed prophylactically in 87 patients (15.1%) and after initiation of RT in 72 patients (12.5%). The number of nonprophylactic feeding tube placements was 59 of 360 patients (16.4%) in those with severe OM and 12 of 216 patients (5.6%) in those without (*P* < .001). The median time to placing a feeding tube was 32 (IQR, 28-43) days from starting RT. Median weight loss for the entire cohort was 4.8 (IQR, 1.8-8.1) kg, and 120 patients (20.8%) required hospitalization. The number of hospitalizations included 90 of 360 patients (25.0%) who developed severe OM and 30 of 216 patients (13.9%) who did not (*P* = .001). Patients were hospitalized at a median of 35 (IQR, 25-45) days from starting RT with a median stay of 4 (IQR, 3-10) days. Opiates were used by 70 patients (12.2%) at the start of RT and 293 (50.9%) by the end of treatment. Opiate use occurred in 224 of 360 patients with severe OM (63%) and 76 of 216 in those without (35%) (*P* < .001). Groups with greater highest severity of OM reported had higher rates of measured outcomes (listed respectively by MTS score 0, 1, 2, 3, and 4): feeding tube placement (0%, 3.6% [2 of 56], 6.6% [10 of 152], 14.7% [40 of 272], and 21.6% [19 of 88]; *P* = .001), hospitalization (12.5% [1 of 8], 10.7% [6 of 56], 15.1% [23 of 152], 23.9% [65 of 272], and 28.4% [25 of 88]; *P* = .02), opiate use (0%, 19.6% [11 of 56], 42.8%[65 of 152], 61.4% [167 of 272], and 64.8% [57 of 88]; *P* < .001) and experienced greater weight loss (median, −0.7 [IQR, −1.7 to −0.4] kg; median, 3.9 [IQR, 1.1 to 6.1] kg; median, 5.0 [IQR, 2.2 to 7.7] kg; median, 4.7 [IQR, 2.1 to 7.7] kg; and median, 7.7 [IQR, 2.8 to 10.6] kg; *P* < .001).

**Table 3.** Prevalence of Selected Clinical Outcomes Grouped by Highest Severity of Patient-Reported MTS During RT for Head and Neck Cancer^a^

Outcome	MTS score^b^					*P* value
	0 (n = 8)	1 (n = 56)	2 (n = 152)	3 (n = 272)	4 (n = 88)	
Feeding tube						
No	5 (62.5)	42 (75)	118 (77.6)	189 (69.5)	64 (72.7)	.001
Yes, before RT	3 (37.5)	12 (21.4)	24 (15.8)	43 (15.8)	5 (5.7)	
Yes, after RT	0 (0)	2 (3.6)	10 (6.6)	40 (14.7)	19 (21.6)	
Hospitalized						
No	7 (87.5)	50 (89.3)	129 (84.9)	207 (76.1)	63 (71.6)	.02
Yes	1 (12.5)	6 (10.7)	23 (15.1)	65 (23.9)	25 (28.4)	
Opiate use						
No	8 (100)	45 (80.4)	87 (57.2)	105 (38.6)	31 (35.2)	<.001
Yes	0 (0)	11 (19.6)	65 (42.8)	167 (61.4)	57 (64.8)	
Weight loss, median (IQR), kg	-0.7 (-1.7 to -0.4)	3.9 (1.1 to 6.1)	5.0 (2.2 to 7.7)	4.7 (2.1 to 7.7)	7.7 (2.8 to 10.6)	<.001

Abbreviations: MTS, mouth and throat soreness; RT, radiation therapy.

^a^ Unless otherwise indicated, data are expressed as No. (%) of patients in each column. Percentages have been rounded and may not total 100.

^b^ Higher scores indicate greater severity.

### QOL Outcomes

Complete baseline and end of RT QOL data were available for 513 patients (89.1%). Across the 6 primary QOL domains evaluated in the European Organisation for Research and Treatment of Cancer Core Quality of Life Questionnaire, mean QOL scores decreased by the end of RT in every domain except for emotional function in those who did not develop severe OM (eTable 2 in Supplement 1). Patients who developed severe OM had worse mean baseline QOL across all reported domains. Mean change in QOL score from the start to end of RT was significantly greater for those who developed severe OM compared with those who did not in the domains of physical function (12.3 [IQR, −26.7 to 0] vs −8.7 [IQR, −20.0 to 0]; *P* = .03), role function (−25.4 [IQR, −50.0 to 0] vs −16.4 [IQR, −33.3 to 0]; *P* = .002), emotional function (−3.3 [IQR, −16.7 to 8.3] vs 1.2 [IQR, −8.3 to 8.3]; *P* = .03), social function (−20.2 [IQR, −50.0 to 0] vs −13.8 [IQR, −33.3 to 0]; *P* = .04), and pain (22.0 [IQR, 0-50.0] vs 16.2 [IQR, 0-33.3]; *P* = .008). No difference in mean QOL change was found for global health (−17.6 [IQR, −33.3 to 0] vs −13.6 [IQR, −33.3 to 0]; *P* = .09) and cognitive function (−7.8 [IQR, −16.7 to 0] vs −5.2 [IQR, −16.7 to 0]; *P* = .25). Patients with severe OM had higher financial difficulty scores at the start and end of RT.

## Discussion

For this large comprehensive study of OM in a contemporary cohort with HNC receiving RT, nearly all patients reported some degree of OM, and 62.5% of patients reported severe OM. Greater severity of OM was associated with feeding tube placement, hospitalization, opiate use, and weight loss. These adverse clinical outcomes contributed to worse QOL and financial difficulties. Continued efforts are needed to improve OM prevention and management.

As expected, the rates of OM in our study were high and progressively worsened during treatment. By the end of RT, 98.6% of patients reported some OM and 62.5% reported severe OM. These rates are consistent with previously published studies of patients treated predominantly with 3-dimensional conformal RT,^3,5,6^ which have reported severe OM in 64% to 81% of patients and some degree of OM in 83% to 98% of patients. Despite known QOL advantages with IMRT planning, previous studies comparing patients treated with IMRT vs non-IMRT planning^4,5^ did not find a statistically significant difference in severe OM rates. This lack of OM reduction may result from the geographic necessity of irradiating mucosa neighboring the tumor regardless of planning technique.

Linear mixed model analysis in our study was unable to identify any disease sites associated with worse OM scores. This is consistent with another study^5^ that found OM to be similar in patients with tumors of the oropharynx or oral cavity compared with tumors of the larynx or hypopharynx. In contrast, other studies^3,6,20^ have identified oropharynx, oral cavity, nasopharynx, and sinonasal sites to be associated with OM. Of these sites, the oropharynx and oral cavity were the most common ones identified. Primary tumors of the oral cavity are likely to have a high RT dose to the oral cavity, and in patients with nasopharyngeal cancer, Orlandi et al^21^ found mean dose to the oral cavity was associated with OM duration. Notably, our study also found bilateral elective neck coverage to be associated with greater severity of OM. This was also demonstrated in a study of oropharynx cancer,^22^ where unilateral IMRT was shown to reduce acute toxic effects of treatment.

Consistent with our findings, 2 previous studies^23,24^ demonstrated smoking to be associated with OM. These findings underscore the importance of smoking cessation prior to initiation of RT for HNC. In addition to worsening OM risk, smoking during treatment has also been linked to worse survival.^25,26^ The contribution of chemotherapy to the development of severe OM remains unclear. While 2 studies^3,6^ demonstrated concurrent chemotherapy to be associated with OM, our study and others^5,20^ did not. We also found no significant difference in OM severity based on chemotherapy regimen; however, patients in our cohort selected for weekly cisplatin typically had worse performance status compared with those receiving high-dose cisplatin. The optimal cisplatin regimen is under investigation.^27^

Multiple clinical outcomes were associated with greater severity of OM, including feeding tube placement, hospitalization, opiate use, and weight loss. Patients who develop OM often have pain with eating and swallowing that can make obtaining adequate nutrition difficult. The rate of nonprophylactic feeding tube placement in our study was 16.4% in those with severe OM and 5.6% in those without. These results are consistent with prior reported rates of 17% to 40% for those who did and 6% to 14% for those who did not develop severe OM.^3,4,5,6^ Given the morbidity of OM symptoms, the association of worsening OM with weight loss was expected and has been demonstrated before.^5,6^ Different criteria for feeding tube placement may be responsible for variations in insertion rates between institutions. Placement of feeding tubes can greatly increase patient financial burden; the average cost to the patient of outpatient percutaneous endoscopic gastrostomy placement can exceed $1100 US dollars.^28,29^

Due to the toxic effects of treatment, patient hospitalization while undergoing RT for HNC is a common occurrence. In our study, severity of OM was associated with hospitalization, and patients with severe OM were twice as likely to be hospitalized (13.9% vs 25.0%). Common reasons for hospitalization in this population include dehydration, malnourishment, and pain control. Other studies^3,6^ have shown similar rates of 23% to 24% and 6% to 16% of patients hospitalized, stratified by development of severe OM. Another report^4^ found that 37% of their cohort of patients with HNC were hospitalized and attributed 30% of the hospitalizations to OM symptoms. Their reported median length of stay of 5 days was consistent with our median stay of 4 days.

Of importance, additional hospitalizations of patients with HNC result in the use of costly resources that can increase the cost of care.^4,6^ In a review of cancer-related oral complications,^30^ the incremental cost of OM was estimated to be approximately $5000 to $7000 per patient. Another study^31^ found the median medical costs for patients to be $39 000 with OM and $21 000 without OM, driven primarily by extended hospitalizations. Consistent with these increased costs, our study found financial difficulty to be higher in patients who developed severe OM. Interestingly, these patients had increased financial difficulty both at baseline and at the end of RT. Identifying better ways to manage OM that decrease the frequency and length of patient hospitalizations can reduce the economic burden contributing to high rates of financial difficulty in this population.^32,33^

Management of OM varies greatly at different institutions. Our institutional standard strongly emphasizes oral hygiene, hydration, and nutrition at the start of treatment and uses prescription medications when necessary as patients develop worsening OM symptoms.^16^ For OM pain control, we prescribe all eligible patients prophylactic gabapentin and recommend alternating between over-the-counter acetaminophen and nonsteroidal anti-inflammatory drugs before prescribing opioids. The use of prophylactic gabapentin is controversial, with multiple studies^34,35,36,37,38^ showing it can reduce and delay opioid use in patients with HNC, while a placebo-controlled randomized clinical trial^39^ found it to have no pain control benefit. Consistent with prior reports,^4,5,6^ we found severity of OM to be associated with opiate use. Patient pain control continues to worsen during treatment despite opioid prescriptions, indicating better solutions are still desperately needed.

For the entire patient cohort, QOL decreased in nearly all domains at the completion of treatment. Despite having worse baseline QOL, patients who developed severe OM experienced a greater QOL decrease in physical, role, emotion, and social function relative to those who did not. Consistent with these results, another study^5^ found worse OM to be associated with reduction in physical and functional well-being. These findings highlight the pervasive effect OM symptoms have on patient QOL. Patients with poor baseline functioning may benefit from additional support to successfully complete treatment.

### Limitations

This study has multiple limitations. All patients were treated by a single radiation oncologist at single academic facility, so outcomes may not be generalizable. Nonacademic centers may not have access to resources such as dedicated staff to administer on-site intravenous fluids as needed, which can significantly affect patient pain control.^40^ This study may also underreport OM symptoms and clinical outcomes due to the exclusion of patients who did not complete RT or have 6 weeks of OM data available. Among the 22 patients not completing RT, 2 experienced progression during treatment, 9 died prior to RT completion, and 11 refused treatment or were hospitalized for an extended period at an outside facility, precluding completion of RT. Complete data on the reason for discontinuing therapy were not available but may have been related to OM symptoms. Other limitations include the absence of additional data that would have made this study more robust, including clinical OM assessment data, patient-reported OM data after treatment completion (follow-up visit), and dosimetric data. Despite these limitations, this study presents comprehensive OM data from a large, uniformly treated patient cohort with rigorous weekly data collection, high survey completion rate, and correlations with QOL data. We hope this report will provide valuable information to help clinicians counsel patients on what symptoms and risks to expect during RT for HNC.

## Conclusions

In this cohort study of patients with HNC treated with IMRT, 62.5% of patients developed severe OM and 98.6% developed some degree of OM. Development of greater-severity OM was associated with feeding tube placement, hospitalization, opiate use, and weight loss. These adverse clinical outcomes contribute to worse QOL and financial difficulty. Further improvements in OM prevention and management are needed.

## Notes

Supplement 1. **eFigure.** Patient CONSORT Diagram **eTable 1.** Patient-Reported Responses to the Oral Mucositis Weekly Questionnaire–Head and Neck Cancer Survey During Radiation Therapy for Head and Neck Cancer **eTable 2.** Mean Patient-Reported EORTC QLQ-C30 Quality of Life Scores at the Start and End of RT Grouped by Development of Severe Oral Mucositis **eAppendix.** Mucositis (Mouth Soreness) Questionnaire

Supplement 2.
Data Sharing Statement
