# Supplementary material for: Severe Oral Mucositis After Intensity-Modulated Radiation Therapy for Head and Neck Cancer
Source: JAMA Netw Open. 2023 Oct 11;6(10):e2337265. doi: 10.1001/jamanetworkopen.2023.37265 (PMC10568356; doi:10.1001/jamanetworkopen.2023.37265)
Supplement: Supplement 1. — eFigure. Patient CONSORT Diagram eTable 1. Patient-Reported Responses to the Oral Mucositis Weekly Questionnaire–Head and Neck Cancer Survey During Radiation Therapy for Head and Neck Cancer eTable 2. Mean Patient-Reported EORTC QLQ-C30 Quality of Life Scores at the Start and End of RT Grouped by Development of Severe Oral Mucositis eAppendix. Mucositis (Mouth Soreness) Questionnaire [file jamanetwopen-e2337265-s001.pdf]

Supplementary Online Content

Iovoli AJ, Turecki L, Qiu ML, et al. Severe oral mucositis after intensity-modulated radiation therapy for head and neck cancer. *JAMA Netw Open*. 2023;6(10):e2337265. doi:10.1001/jamanetworkopen.2023.37265

eFigure. Patient CONSORT Diagram

eTable 1. Patient-Reported Responses to the Oral Mucositis Weekly Questionnaire–Head and Neck Cancer Survey During Radiation Therapy for Head and Neck Cancer

eTable 2. Mean Patient-Reported EORTC QLQ-C30 Quality of Life Scores at the Start and End of RT Grouped by Development of Severe Oral Mucositis

eAppendix. Mucositis (Mouth Soreness) Questionnaire

This supplementary material has been provided by the authors to give readers additional information about their work.

© 2023 Iovoli AJ et al. *JAMA Network Open*.

eFigure. Patient CONSORT Diagram

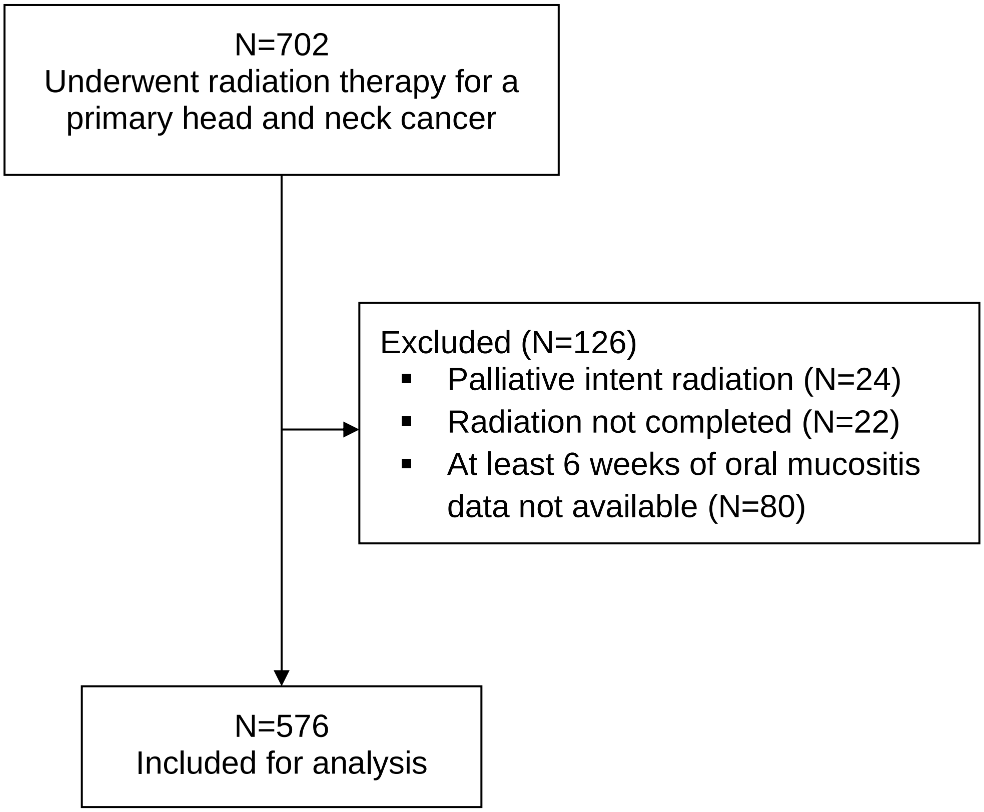

© 2023 Iovoli AJ et al. *JAMA Network Open*.

eTable 1. Patient-Reported Responses to the Oral Mucositis Weekly Questionnaire–Head and Neck Cancer Survey During Radiation Therapy for Head and Neck Cancer

	Week of Radiation Therapy													
	**1**		**2**		**3**		**4**		**5**		**6**		**7**	
**MTS**														
No Soreness	369	64.1%	256	44.4%	61	10.6%	54	9.4%	55	9.5%	38	6.6%	26	5.6%
A Little Soreness	139	24.1%	198	34.4%	177	30.7%	174	30.2%	144	25.0%	120	20.8%	89	19.1%
Moderate Soreness	43	7.5%	89	15.5%	180	31.3%	186	32.3%	200	34.7%	206	35.8%	145	31.1%
Quite a lot of Soreness	20	3.5%	30	5.2%	137	23.8%	145	25.2%	149	25.9%	173	30.0%	166	35.6%
Extreme Soreness	5	0.9%	3	0.5%	21	3.6%	17	3.0%	28	4.9%	38	6.6%	40	8.6%
**Swallowing**														
Not limited	460	79.9%	424	73.6%	184	31.9%	184	31.9%	159	27.6%	129	22.4%	86	18.5%
Limited a little	60	10.4%	84	14.6%	146	25.3%	147	25.5%	131	22.7%	119	20.7%	98	21.0%
Limited some	25	4.3%	35	6.1%	134	23.3%	135	23.4%	135	23.4%	146	25.3%	105	22.5%
Limited a lot	17	3.0%	16	2.8%	96	16.7%	93	16.1%	130	22.6%	153	26.6%	154	33.0%
Unable to do	14	2.4%	17	3.0%	16	2.8%	17	3.0%	21	3.6%	28	4.9%	23	4.9%
**Drinking**														
Not limited	500	86.8%	481	83.5%	296	51.4%	254	44.1%	227	39.4%	190	33.0%	119	25.5%
Limited a little	30	5.2%	49	8.5%	119	20.7%	146	25.3%	120	20.8%	105	18.2%	101	21.7%
Limited some	14	2.4%	17	3.0%	85	14.8%	88	15.3%	110	19.1%	140	24.3%	103	22.1%
Limited a lot	9	1.6%	12	2.1%	55	9.5%	67	11.6%	97	16.8%	105	18.2%	110	23.6%
Unable to do	23	4.0%	17	3.0%	21	3.6%	21	3.6%	22	3.8%	35	6.1%	33	7.1%
**Eating**														
Not limited	458	79.5%	399	69.3%	169	29.3%	129	22.4%	125	21.7%	97	16.8%	55	11.8%
Limited a little	49	8.5%	78	13.5%	123	21.4%	139	24.1%	100	17.4%	78	13.5%	68	14.6%
Limited some	21	3.6%	53	9.2%	132	22.9%	140	24.3%	121	21.0%	116	20.1%	95	20.4%
Limited a lot	16	2.8%	18	3.1%	117	20.3%	129	22.4%	178	30.9%	209	36.3%	170	36.5%
Unable to do	32	5.6%	28	4.9%	35	6.1%	39	6.8%	52	9.0%	75	13.0%	78	16.7%
**Talking**														
Not limited	461	80.0%	447	77.6%	358	62.2%	309	53.6%	286	49.7%	235	40.8%	148	31.8%

© 2023 Iovoli AJ et al. *JAMA Network Open*.

Limited a little	45	7.8%	67	11.6%	95	16.5%	129	22.4%	105	18.2%	129	22.4%	107	23.0%
Limited some	32	5.6%	27	4.7%	67	11.6%	80	13.9%	104	18.1%	116	20.1%	105	22.5%
Limited a lot	19	3.3%	14	2.4%	37	6.4%	34	5.9%	66	11.5%	74	12.8%	87	18.7%
Unable to do	19	3.3%	21	3.6%	19	3.3%	24	4.2%	15	2.6%	21	3.6%	19	4.1%
**Sleeping**														
Not limited	483	83.9%	471	81.8%	392	68.1%	394	68.4%	360	62.5%	326	56.6%	231	49.6%
Limited a little	36	6.3%	48	8.3%	93	16.1%	60	10.4%	80	13.9%	99	17.2%	77	16.5%
Limited some	36	6.3%	36	6.3%	50	8.7%	81	14.1%	81	14.1%	73	12.7%	85	18.2%
Limited a lot	20	3.5%	20	3.5%	40	6.9%	39	6.8%	55	9.5%	76	13.2%	73	15.7%
Unable to do	1	0.2%	1	0.2%	1	0.2%	2	0.3%	0	0.0%	1	0.2%	0	0.0%
**Food Consumption**														
Normal diet	381	66.1%	323	56.1%	122	21.2%	81	14.1%	53	9.2%	44	7.6%	30	6.4%
Restricted diet	96	16.7%	147	25.5%	258	44.8%	232	40.3%	190	33.0%	137	23.8%	78	16.7%
Liquid & pureed only	30	5.2%	34	5.9%	123	21.4%	179	31.1%	241	41.8%	269	46.7%	256	54.9%
PEG tube & some oral	38	6.6%	38	6.6%	40	6.9%	52	9.0%	48	8.3%	60	10.4%	39	8.4%
PEG tube only	31	5.4%	34	5.9%	33	5.7%	32	5.6%	44	7.6%	65	11.3%	63	13.5%
**Mean Overall Health Rating**	7.75		7.56		7.06		6.81		6.33		6.18		5.91	
**Mean MTS Rating**	1.44		2.01		4.19		4.46		4.53		5.04		5.38	

MTS: Mouth and throat soreness.

© 2023 Iovoli AJ et al. *JAMA Network Open*.

eTable 2. Mean Patient-Reported EORTC QLQ-C30 Quality of Life Scores at the Start and End of RT Grouped by Development of Severe Oral Mucositis

	**Non-Severe OM (n=199)**			**Severe OM (n=314)**			
**QOL Measure**	**RT Start**	**RT End**	**Mean Change (IQR)**	**RT Start**	**RT End**	**Mean Change (IQR)**	**p-value**
Global Health	76.2	62.6	-13.6 (-33.3-0)	68.3	50.7	-17.6 (-33.3-0)	0.09
Physical Function	89.9	81.1	-8.7 (-20.0-0)	84.9	72.5	-12.3 (-26.7-0)	0.03
Role Function	85.7	69.3	-16.4 (-33.3-0)	79.5	54.1	-25.4 (-50.0-0)	0.002
Emotional Function	82.0	83.2	1.2 (-8.3-8.3)	71.7	68.4	-3.3 (-16.7-8.3)	0.03
Cognitive Function	90.9	85.7	-5.2 (-16.7-0)	82.6	74.8	-7.8 (-16.7-0)	0.25
Social Function	84.7	70.9	-13.8 (-33.3-0)	77.9	57.7	-20.2 (-50.0-0)	0.04
Fatigue	79.5	58.4	-21.1 (-33.3-0)	70.4	44.8	-25.6 (-44.4-11.1)	0.08
Pain	15.0	31.2	16.2 (0-33.3)	31.5	53.5	22.0 (0-50.0)	0.008
Insomnia	22.8	29.6	6.8 (0-33.3)	36.2	42.6	6.4 (-33.3-33.3)	0.96
Financial Difficulty	13.6	17.3	3.7 (0-0)	24.9	26.5	1.6 (0-0)	0.35

OM: oral mucositis; QOL: quality of life; IQR: interquartile range.

The modified Oral Mucositis Weekly Questionnaire-Head and Neck Cancer (OMWQ-HN) survey used in our REDCap database is shown in the following two pages.

© 2023 Iovoli AJ et al. *JAMA Network Open*.

eAppendix. Mucositis (Mouth Soreness) Questionnaire

Please complete the survey below.

Thank you!

Please enter your Medical Record Number

____

How would you rate your OVERALL HEALTH during the LAST 24 HOURS?

0 - Worst Possible	5	10 - Perfect Health

(Place a mark on the scale above)

During the LAST 24 HOURS, how much MOUTH AND THROAT SORENESS did you have?

- ◯ No Soreness
- ◯ A Little Soreness
- ◯ Moderate Soreness
- ◯ Quite a lot of Soreness
- ◯ Extreme Soreness

© 2023 Iovoli AJ et al. *JAMA Network Open*.

During the PAST 24 HOURS, how much did MOUTH AND THROAT SORENESS limit you in each of the following activities?

	Not Limited	Limited A Little	Limited Some	Limited A Lot	Unable To Do
Swallowing	◯	◯	◯	◯	◯
Drinking	◯	◯	◯	◯	◯
Eating	◯	◯	◯	◯	◯
Talking	◯	◯	◯	◯	◯
Sleeping	◯	◯	◯	◯	◯

On a scale of 1 to 10, how would you rate your OVERALL MOUTH AND THROAT SORENESS during the LAST 24 hours

0, No Soreness — 5 — 10, Worst Possible

(Place a mark on the scale above)

What food are you consuming orally?

- ◯ I can eat anything I want (Normal diet).
- ◯ I can eat most foods but have started to avoid some (for example too hard, too chewy, or too spicy)
- ◯ I can only consume liquids and pureed foods.
- ◯ I am using my PEG tube and trying to eat a few things also.
- ◯ I am using my PEG tube only.

How many cans on PEG feeding are you using?

◯ 1 ◯ 2 ◯ 3 ◯ 4 ◯ 5 ◯ 6 ◯ 7 ◯ 8

What type of PEG Feeding?

- ◯ Boost Plus
- ◯ Ensure Plus
- ◯ Jevity
- ◯ Glucema
- ◯ Isosource
- ◯ Other

What other type(s) of PEG feeding are you using?

____

© 2023 Iovoli AJ et al. *JAMA Network Open*.
