# Supplementary material for: Severe Oral Mucositis After Intensity-Modulated Radiation Therapy for Head and Neck Cancer
Source: JAMA Netw Open. 2023 Oct 11;6(10):e2337265. doi: 10.1001/jamanetworkopen.2023.37265 (PMC10568356; doi:10.1001/jamanetworkopen.2023.37265)
Supplement: Supplement 2. — Data Sharing Statement [file jamanetwopen-e2337265-s002.pdf]

Data Sharing Statement

Iovoli. Severe Oral Mucositis After Intensity-Modulated Radiation Therapy for Head and Neck Cancer. *JAMA Netw Open*. Published October 11, 2023.
doi:10.1001/jamanetworkopen.2023.37265

Data
Data available: No

Additional Information
Explanation for why data not available: Research data are stored in an institutional repository and will be shared upon request to the cor-responding author.
